# Rheology of α-Gel Formed by Amino Acid-Based Surfactant with Long-Chain Alcohol: Effects of Inorganic Salt Concentration

**DOI:** 10.1021/acs.langmuir.1c00626

**Published:** 2021-06-03

**Authors:** Kumika Ichihara, Tadashi Sugahara, Masaaki Akamatsu, Kenichi Sakai, Hideki Sakai

**Affiliations:** † Department of Pure and Applied Chemistry, Faculty of Science and Technology, Tokyo University of Science, 2641 Yamazaki, Noda, Chiba 278-8510, Japan; ‡ Research Institute for Science and Technology, Tokyo University of Science, 2641 Yamazaki, Noda, Chiba 278-8510, Japan

## Abstract

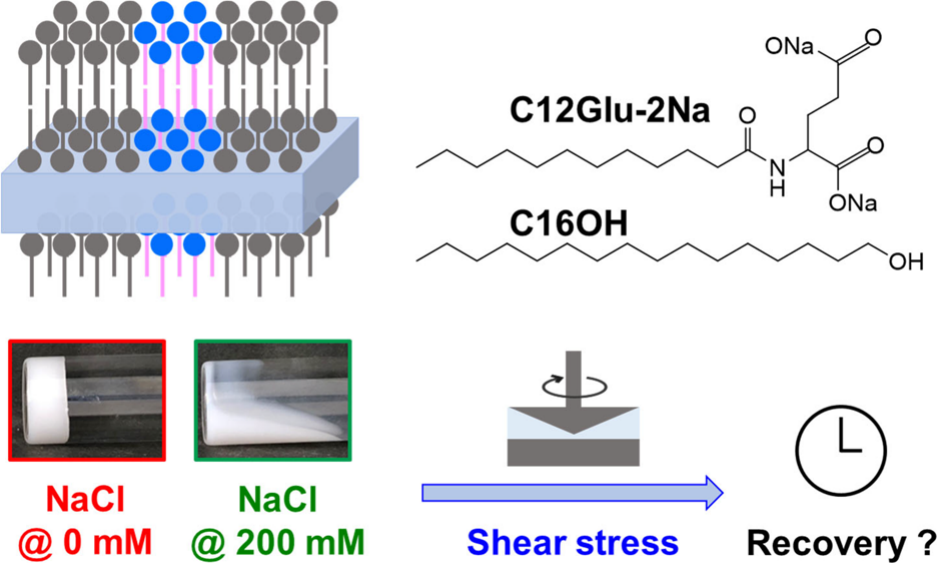

Mixtures of surfactants, long-chain alcohols, and water sometimes yield lamellar gels with hexagonally packed alkyl chains. This assembly is called “α-gel” or “α-form hydrated crystal.” In this study, we characterized the rheological properties of α-gel prepared using disodium *N*-dodecanoylglutamate (C12Glu-2Na), 1-hexadecanol (C16OH), and water at different NaCl concentrations. The α-gel structure was assessed using small- and wide-angle X-ray scattering (SWAXS). The SWAXS measurements revealed that an increased NaCl concentration (0–200 mmol dm^–3^) resulted in a decreased *d*-spacing caused by the screening of electrostatic repulsion between lamellar bilayers. This led to an increased amount of excess water (i.e., the water present between the α-gel domains), and hence, the viscosity of the α-gel decreased in the range of the NaCl concentration. A further increase in the NaCl concentration (200–1000 mmol dm^–3^) resulted in decreased electrostatic repulsion between the α-gel domains and/or an increased number of α-gel domains (multilamellar vesicles). These effects increased the domain-to-domain interactions, leading to increased viscosity. Therefore, we concluded that the viscosity of the α-gel was controlled by the amount of excess water and the domain-to-domain interactions. Once the network structure collapsed under the strain, it was difficult to recover the original network structure. The low recoverability resulted from increased cohesion between the domains at high NaCl concentrations.

## Introduction

Binary mixtures of surfactants and water or ternary mixtures of surfactants, long-chain alcohols, and water sometimes yield “lamellar gel” below their gel–liquid crystal phase transition temperatures (*T_c_*). The molecular assembly called “α-gel” or “α-form hydrated crystal” is a type of lamellar gel. Structurally, the alkyl chains of the surfactant and long-chain alcohol forming the α-gel are hexagonally packed within the lamellar bilayers below its *T_c_*,^1−3^ while those forming the lamellar liquid crystal are in a molten state above *T_c_*. The α-gel can hold a large amount of water in its lamellar structure and the spaces between domains and generally yields highly viscous creams.^4,5^ Therefore, α-gel has been widely used in personal care products, such as shampoos, hair conditioners, and skincare creams.^6−12^

The rheological properties of α-gel systems have been widely studied,^13−16^ and their active control is required. Additionally, the term “psycho-rheology” denotes the importance of rheological usability in such applications.^17−19^ Yamagata and coworkers^20−24^ discussed the rheological behavior of the ternary mixture of hexadecyltrimethylammonium chloride, 1-hexadecanol (C16OH), and water based on electron microscopy and electron spin resonance data. Their key finding was that the viscosity of the α-gel consisting of a lamellar network structure was higher than that of an α-gel consisting of multilamellar vesicular domains. The former structure was prepared at a mixing temperature below its *T_c_*, while the latter domains were prepared at a mixing temperature above its *T_c_*. Similarly, Nakagawa and coworkers^25^ studied the rheology of a mixed system of distearoyl phosphatidylcholine (DSPC), distearoyl phosphatidylglycerol (DSPG), C16OH, and water. They found that an increased mole content of DSPG changed the morphology of the α-gel domains from a network to vesicles, leading to a decreased viscosity. Recently, we reported that an increased domain size led to the increased viscosity of the α-gel formed by a mixture of *N*-[3-(dimethylamino)propyl] docosanamide l-lactic acid salt, 1-octadecanol (C18OH), and water.^26^ These earlier works suggest that the shape and size of the α-gel domains have a significant impact on the viscosity. For the mechanical properties, the viscosity of the lamellar gel network (α-gel) formed by docosyltrimethylammonium methylsulfate was reported to be higher than that by docosyltrimethylammonium chloride because the network in the methylsulfate system was considerably stiff.^10^

Inorganic salts are frequently formulated as viscosity modifiers in personal care cream products. Additionally, the applied skincare creams may interact with the salts dissolved in sweat. Therefore, the effect of inorganic salts on the viscosity of α-gel is an important subject in academia and the industry.^11,27,28^ Eccleston and coworkers^7^ reported that the addition of NaCl to a mixture of surfactants (dodecyltrimethylammonium bromide, tetradecyltrimethylammonium bromide, and hexadecyltrimethylammonium bromide), long-chain alcohols (C16OH and C18OH), and water resulted in decreased viscosity, leading to the destabilization of the α-gel system (i.e., the separation of water from the cream phase). Recently, it was found that the addition of CaCl_2_ into a mixture of a double-chain cationic surfactant and water induces a structural change in the α-gel domains from layered lamellae to multilamellar vesicles, leading to decreased viscosity.^29^

The earlier works mentioned above, including our previous works, demonstrated that the rheological properties of α-gel are significantly affected by the shape and size of the α-gel domains. In other words, it is important to elucidate domain-to-domain interactions in α-gel systems for understanding their rheological properties. Particularly, the domain-to-domain interactions will be crucial in a process of structural recovery of α-gel after an input of shear stress. Nevertheless, the number of studies focusing on this topic is limited in terms of background electrolyte concentration. The goal of this study is to propose domain-to-domain interaction models, based on static and dynamic rheological data. The α-gel was prepared using an amino-acid-based surfactant (disodium *N*-dodecanoylglutamate (C12Glu-2Na)), C16OH, and water at different NaCl concentrations.

## Materials and Methods

### Materials

*N*-Dodecanoylglutamic acid (C12Glu) was synthesized according to the procedure described in our previous papers.^30,31^ C12Glu was neutralized using NaOH at a fixed mole ratio, C12Glu:NaOH = 1:2, to yield C12Glu-2Na. C16OH and NaCl were purchased from FUJIFILM Wako Pure Chemical Corporation and used without further purification. The water used in this study was purified using a Millipore Direct-Q UV3 system.

### Sample Preparation

C12Glu-2Na, C16OH, and NaCl aqueous solutions were mixed in a glass vial. The mixture was heated at 80 °C for 1 h in a temperature-controlled water bath. This temperature was much higher than the melting point of C16OH in its hydrated state (52.1 °C).^32^ Subsequently, the samples were stirred using a vortex mixer at 3000 rpm for 3 min. This heating–stirring cycle was repeated three times. After allowing the mixture to stand at 25 °C for 1 h, degassing from each viscous sample was performed using a Kokusan H-28F centrifuge at a constant rotation speed of 2000 rpm for 5 min. The water concentration was always set at 90 wt % under a given mole ratio of C12Glu-2Na:C16OH = 1:3. NaCl was added in the concentration range of 0–1000 mmol dm^–3^.

### Methods

Small- and wide-angle X-ray scattering (SWAXS) measurements were performed using an Anton Paar SAXSess instrument. The apparatus was operated at 40 kV and 50 mA using a line-collimated Cu Kα X-ray source (wavelength = 0.154 nm). The X-ray irradiation time was fixed at 20 min.

Rheological measurements were performed using an Anton Paar MCR302 rheometer. We used cone–plate geometries (diameter = 25 mm, cone angle = 2°). All the rheological measurements were performed for 5 min after setting the samples. The static and dynamic rheological measurements were performed repeatedly at least three (static) or two (dynamic) times for each data point.

All measurements were performed at 25 °C, 1 day after the sample preparation.

## Results and Discussion

### Characterization of α-Gel Structure

**Figure 1:**
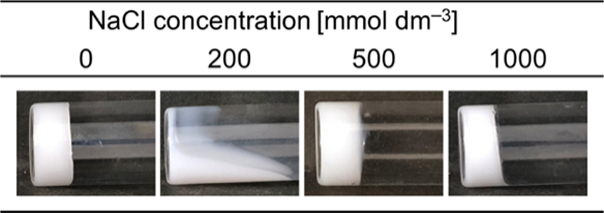
Visual appearance of the samples prepared at different NaCl concentrations.

Figure 1 shows the visual appearance of the samples prepared at different NaCl concentrations. The prepared samples were white and highly viscous. Fluidity was only observed for the sample prepared using 200 mmol dm^–3^ of NaCl. In the next section, we discuss the rheology of these samples in detail.

**Figure 2:**
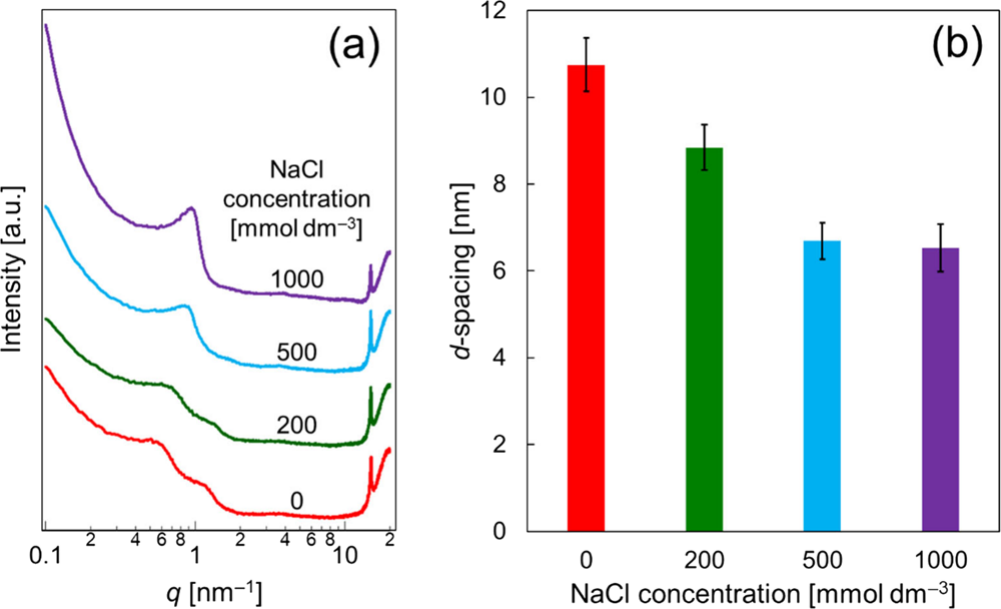
(a) SWAXS patterns obtained for the samples prepared at different NaCl concentrations and (b) *d*-spacing calculated from the *q*_1_ values in (a). The error bars shown in (b) were calculated using individual three repeated data.

Here, we discuss the formation of α-gels for these samples. The SWAXS patterns obtained for these samples are shown in Figure 2a. Repeated broad peaks with a scattering vector (*q*) ratio of 1:2 were observed at [NaCl] = 0 and 200 mmol dm^–3^ in the small-angle region. These peaks indicate the formation of a lamellar structure. Furthermore, a sharp peak was observed at 15 nm^–1^, indicating the hexagonal packing of the alkyl chains. These results confirm the formation of the α-gel, as previously reported for the NaCl-free α-gel system.^33^ The first peak became sharper as the NaCl concentration increased; however, the second scattering peak in the small-angle region disappeared (or significantly broadened) at [NaCl] = 500 and 1000 mmol dm^–3^. Although the exact reason for this is presently unclear, a change in the intermembrane interaction may contribute to the disappearance.^29^

The scattering vector corresponding to the first peak (*q*_1_) in the small-angle region was used to calculate the lamellar *d*-spacing according to the following eq 1:
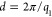
1

The calculated lamellar *d*-spacing values are plotted in Figure 2b. The increased NaCl concentration resulted in an increased *q*_1_ (Figure 2a), thereby decreasing the *d*-spacing (Figure 2b). The increased NaCl concentration screened the electrostatic repulsion between bilayers, forming the α-gel structure. In other words, the increased NaCl concentration afforded a decreased osmotic pressure from the charged headgroups (or from the charged bilayers). The importance of the electrostatic interaction on the lamellar *d*-spacing was similarly suggested by Eccleston et al.^7^ and Yanase et al.^29^ in their lamellar gel systems. The addition of salting-out salts also induced the dehydration of the charged headgroups, as previously suggested in lamellar liquid crystal systems.^34,35^ These effects necessarily contribute to the decreased *d*-spacing caused by the decreased thickness of the water phase sandwiched between bilayers.

### Rheological Behavior

**Figure 3:**
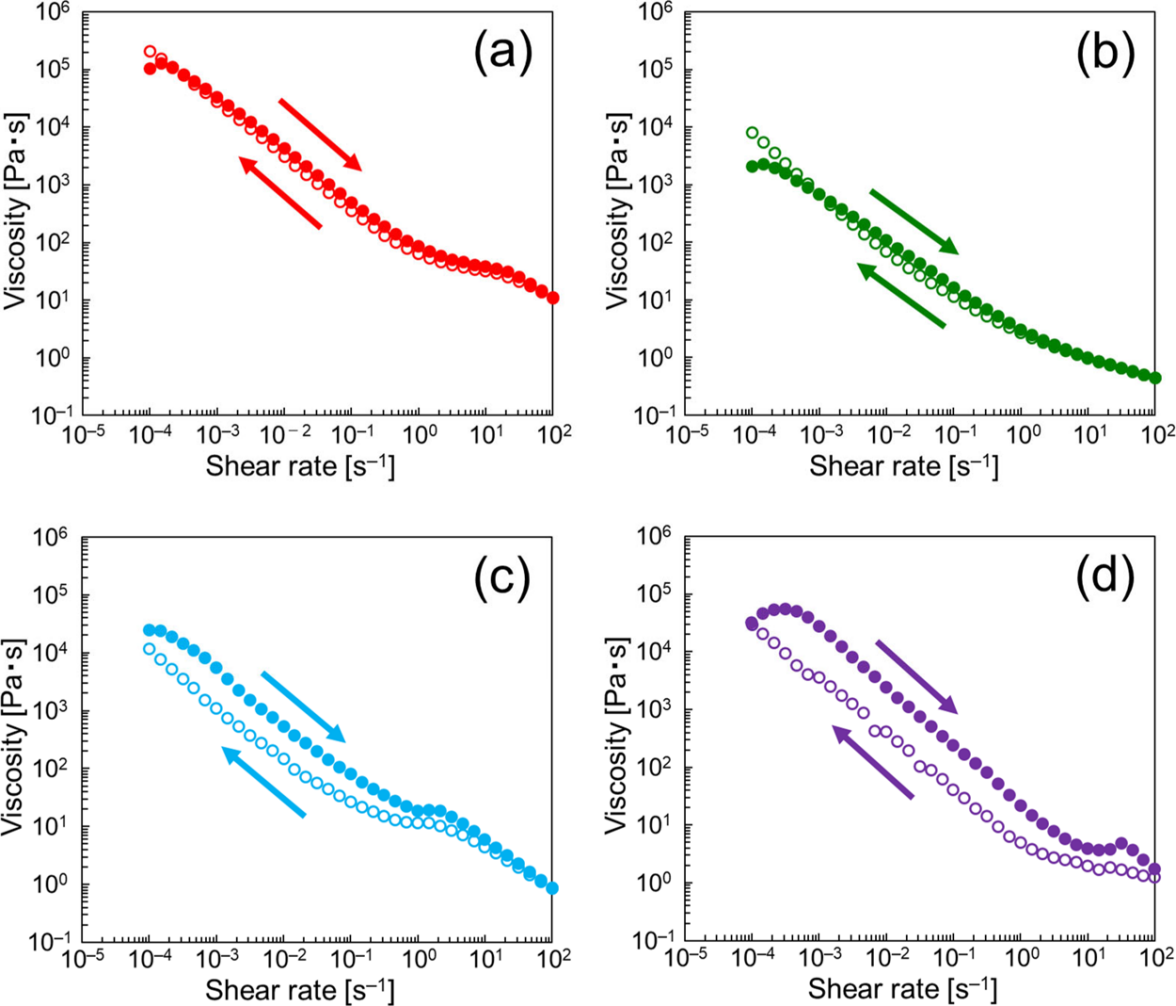
Static viscosity as a function of shear rate. The NaCl concentrations were varied at (a) 0, (b) 200, (c) 500, and (d) 1000 mmol dm^–3^. The filled circles correspond to the first shear rate sweep at increasing shear rates, while the empty circles correspond to the second shear rate sweep at decreasing shear rates.

As mentioned in the Introduction, the α-gel is widely used as a base for cream products. Therefore, it is necessary to understand and control its rheology to improve its usability. Figure 3 shows the static viscosity in the shear rate range of 1 × 10^–4^–1 × 10^2^ s^–1^. Viscosity was measured in two consecutive shear rate sweeps: the first one at an increasing shear rate, and the second one at a decreasing shear rate. At all NaCl concentrations examined, the viscosity decreased sharply with the increasing shear rate. This confirms the shear thinning of the α-gel samples.

One may notice the presence of inflection points in the first and second sweeps in the shear rate range of approximately 2 × 10^0^–3 × 10^1^ s^–1^. Similar inflection points were observed previously in the α-gel system.^33^ Recently, we performed simultaneous small-angle neutron scattering and rheological (Rheo-SANS) measurements in an α-gel system consisting of an anionic gemini surfactant, 1-tetradecanol, and water.^36^ In this earlier work, we found that the bilayer structure remained even at high shear rates.^36^ It seems, therefore, that the inflection points result from a change in the domain-to-domain network structure, although the exact mechanism for the observed behavior is not clear at present.

**Figure 4:**
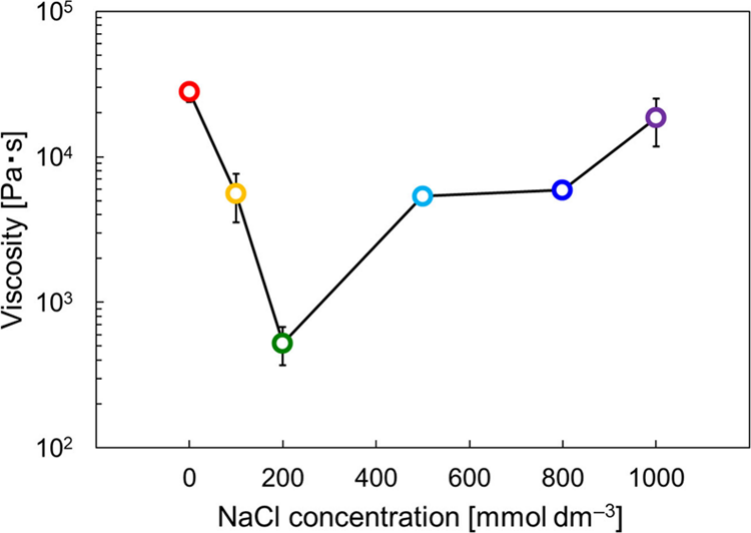
Viscosity measured at the shear rate of 1 × 10^–3^ s^–1^ in the first increasing shear rate sweep at different NaCl concentrations. The viscosities measured at [NaCl] = 100 and 800 mmol dm^–3^ were also plotted in this figure. The corresponding static viscosity data are given in the Supporting Information (Figure S2). The error bars were calculated using individual three repeated data.

The viscosity measured at a shear rate of 1 × 10^–3^ s^–1^ in the first increasing shear rate sweep was plotted against the NaCl concentration, as shown in Figure 4. Due to the increased NaCl concentration, the viscosity decreased at 200 mmol dm^–3^, and afterward, it increased to 1000 mmol dm^–3^. The presence of a minimum viscosity as a function of inorganic salt concentration was similarly reported for a mixed system of double-chained cationic surfactant, water, and CaCl_2_.^29^ In our previous work,^37^ we proposed two important factors that determine the viscosity of the α-gel. The first factor was the amount of excess water (i.e., the presence of water between the α-gel domains^38,39^). As mentioned in the previous section, the increased NaCl concentration resulted in a decreased *d*-spacing, and hence, a decreased amount of water sandwiched between bilayers. This leads to an increased amount of excess water. Therefore, the decreased viscosity observed in the [NaCl] range of 0–200 mmol dm^–3^ possibly resulted from the increased amount of excess water. The second factor was the morphology of the α-gel domains, i.e., the viscosity decreased when a lamellar network structure transformed into an onion structure. We performed optical and polarized microscopies to determine the domain structure of the α-gel in the present system. The results are shown in the Supporting Information (Figure S1). A multilamellar vesicle or onion-like domain structure was confirmed at the NaCl concentrations investigated in this study. However, their size did not change within the resolution of our instrument. Therefore, we suggest that the domain size had little impact on the increased viscosity observed above [NaCl] = 200 mmol dm^–3^.

Figure 3 also demonstrates the thixotropy (i.e., viscosity hysteresis) for these samples. This thixotropic behavior was particularly obvious at NaCl concentrations of 500 and 1000 mmol dm^–3^, suggesting that the structural recovery of the domain-to-domain networks was significantly delayed at these NaCl concentrations.

**Figure 5:**
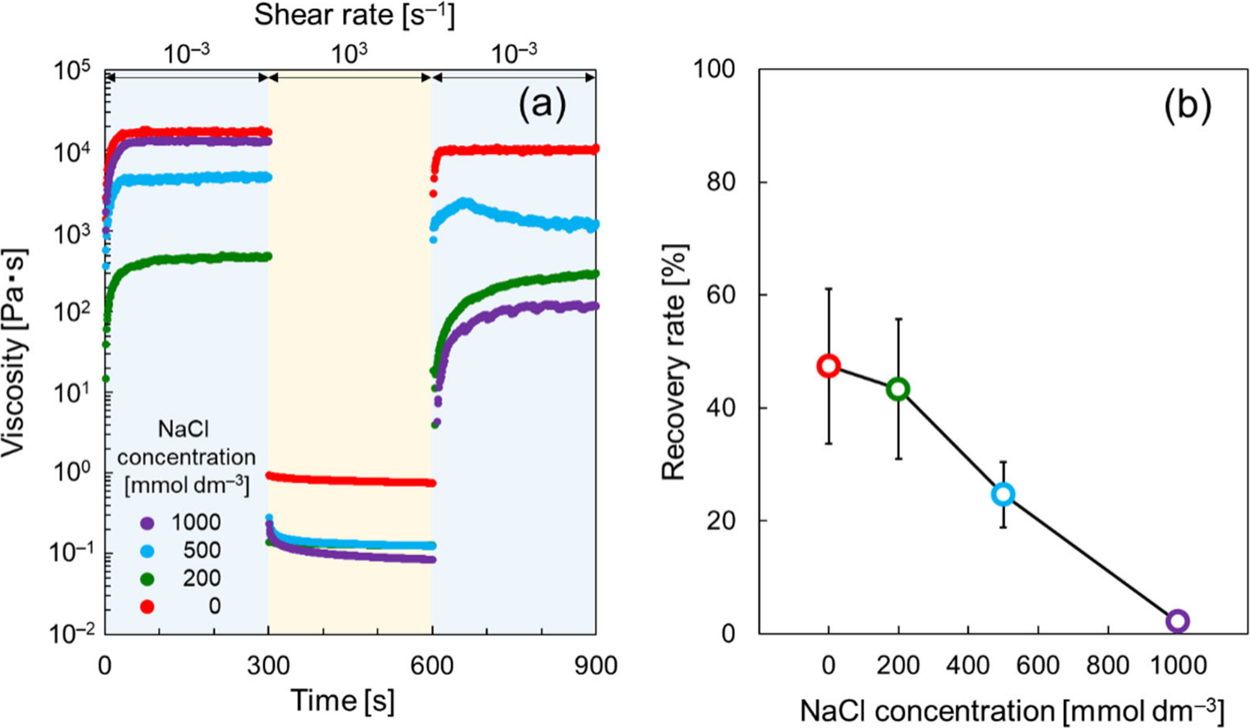
(a) Stepwise measurements of viscosities at different NaCl concentrations. (b) Recovery rate estimated at 1 × 10^–3^ and 1 × 10^3^ s^–1^ based on the data shown in (a). The error bars shown in (b) were calculated using individual three repeated data.

The thixotropic behavior was further analyzed using the following stepwise measurements. Figure 5a shows the viscosity measured at the two shear rates (1 × 10^–3^ and 1 × 10^3^ s^–1^) in the following steps: 1 × 10^–3^ s^–1^ → 1 × 10^3^ s^–1^ → 1 × 10^–3^ s^–1^. The viscosity decreased sharply when the shear rate increased to 1 × 10^3^ s^–1^, and afterward, it increased as the shear rate decreased to 1 × 10^–3^ s^–1^. Importantly, the viscosity did not completely return to the original level, particularly at high NaCl concentrations. The recovery rate,^40^ defined as the equilibrium viscosity measured in the third step at 900 s divided by that in the first step at 300 s, was calculated at different NaCl concentrations. Figure 5b shows the results of the calculations. The increased NaCl concentration resulted in a decreased recovery rate of the domain-to-domain network structure. It seems likely that the increased NaCl concentration induces the decreased electrostatic repulsion between domains and/or the dehydration of each domain. These effects necessarily contribute to the decreased recovery rate observed at high NaCl concentrations, accompanying with increased cohesion between the domains.

**Figure 6:**
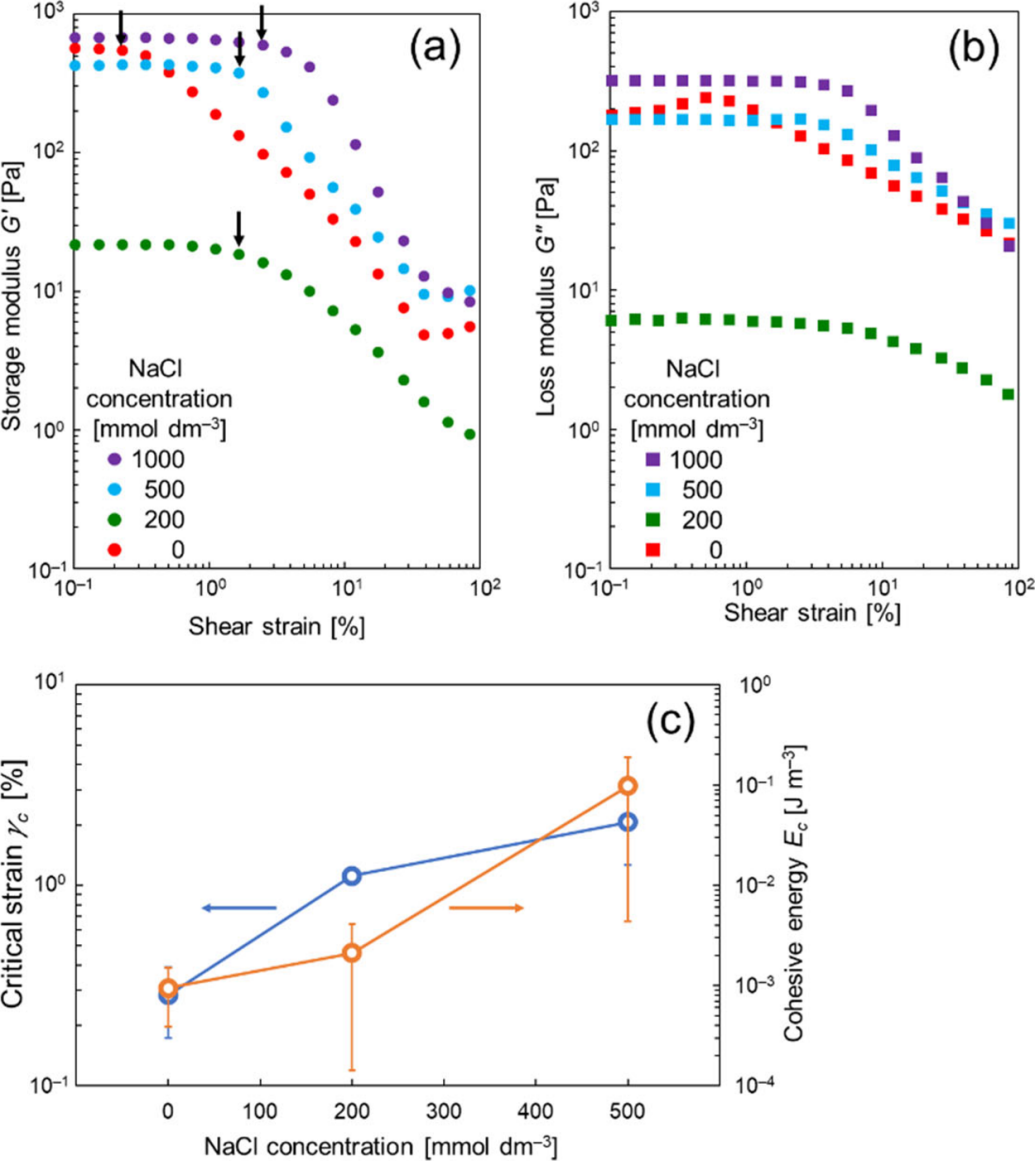
(a) Storage modulus *G*′ and (b) loss modulus *G*″ of the samples prepared at different NaCl concentrations. (c) Critical strain γ*_c_* and cohesive energy *E_c_* calculated using eq 2. The arrows in (a) show the critical strains (γ*_c_*). In these measurements, the angular frequency was always set at 1 rad s^–1^. The torque was measured as above 0.1 μN m (the lower limit torque of our instrument), and hence the measurements were performed properly for the data points shown in this figure. The error bars shown in (c) were calculated using individual two repeated data. The γ*_c_* value measured at [NaCl] = 1000 mmol dm^–3^ was scattered, and hence the γ*_c_* and *E_c_* values at this NaCl concentration were not shown in (c).

The domain-to-domain interactions were studied using dynamic viscosity measurements. Figure 6 shows the dynamic viscoelasticity of the α-gel samples measured in the shear strain range of 1 × 10^–2^–1.2 × 10^2^ %: (a) storage modulus *G*′ and (b) loss modulus *G*″. *G*′ was constant at low shear strains, and afterward, it decreased sharply from a critical strain (γ*_c_*). This suggests that the deformation of the internal structures constructed by the agglomeration of domains occurs above the shear strain.^41,42^Figure 6c presents the γ*_c_* as a function of NaCl concentration. The increased NaCl concentration resulted in increased γ*_c_*, suggesting that the resistance of the internal structure (i.e., the domain-to-domain network structure) to strain increased with increasing NaCl concentration. The increased NaCl concentration also yielded low *G*′ values at 200 mmol dm^–3^ (shear strain < γ*_c_*), followed by high *G*′ values at 500 and 1000 mmol dm^–3^. This order is consistent with that of the viscosity, as shown in Figure 4, and that of *G*″ at low shear strains (<γ*_c_*), as shown in Figure 6b.

The cohesive energy, *E_c_*, is the energy required for the domain-to-domain network structure to initiate a transition.^43^ The *E_c_* can be calculated using eq 2,^24,43^ where σ is the stress applied to the sample, and γ is the shear strain.
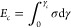
2

The calculated *E_c_* values are shown in Figure 6c. Although the error bars were somewhat overlapped at different NaCl concentrations, the *E_c_* tended to increase with NaCl concentration. This indicates that the domain-to-domain interactions increased as the NaCl concentration increased. Although the amount of excess water increased with increasing NaCl concentration, the *E_c_* drastically increased at [NaCl] = 500 mmol dm^–3^ when the viscosity increased. Therefore, the viscosity of our α-gel samples is determined not only by the amount of excess water but also by the domain-to-domain network structure to strain. The amount of excess water predominantly affected the viscosity at [NaCl] = 0–200 mmol dm^–3^, while the domain-to-domain interactions contributed to the increased viscosity at 200 mmol dm^–3^ < [NaCl]. The addition of NaCl caused the increased resistance to strain, leading to the difficulty in recovering the original network structure once the structure collapsed under strain (see Figures 5 and 6).

**Figure 7:**
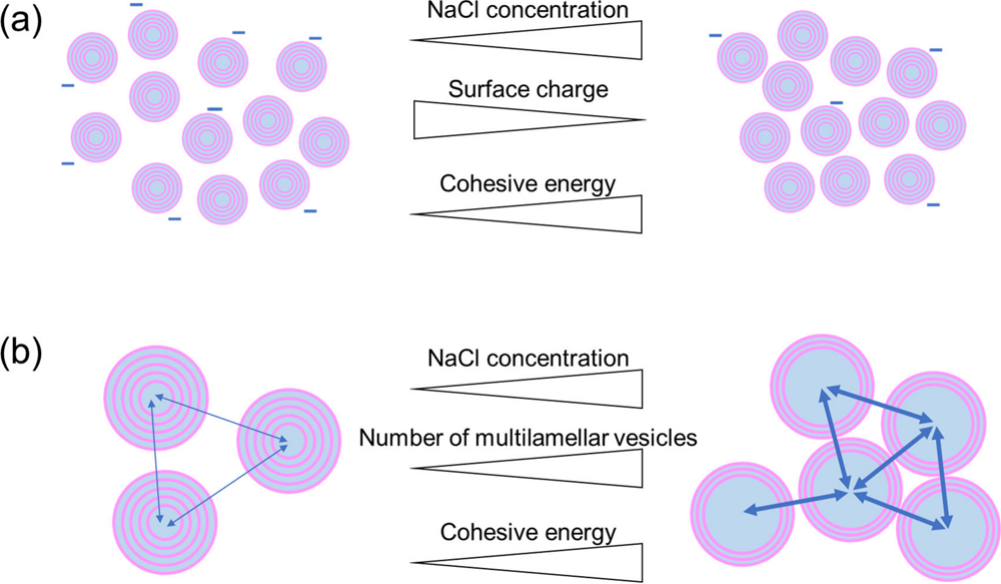
Schematic illustration for the domain-to-domain interactions at different NaCl concentrations. The increased cohesive energy at high NaCl concentration results from (a) the decreased electrostatic repulsion between the domains and/or (b) the increased number of multilamellar vesicles.

We hypothesize two factors affecting the domain-to-domain interactions. The first factor is the change in the electrostatic repulsion between the domains (i.e., multilamellar vesicles or onions) at different NaCl concentrations. The addition of NaCl screens the surface charges on the domains. This necessarily results in an increased domain-to-domain interaction, and therefore, *E_c_* increases at high NaCl concentrations. Additionally, the addition of NaCl results in the dehydration of each domain, leading to the increased *E_c_*. The other factor we consider is that the increased NaCl concentration results in an increased number of multilamellar vesicles. This model was reported in the system of double-chained cationic surfactant, water, and CaCl_2_.^29^ This model predicts that the excess water at high NaCl concentrations is incorporated into the core of the multilamellar vesicles, accompanied by an increased number of vesicles with thinner shells. In this situation, the distance between the vesicles decreases, and hence, their interaction (i.e., *E_c_*) increases at high NaCl concentrations. In our case, the size of the domains (or multilamellar vesicles) did not change significantly within the resolution (Figure S1), being similar to the case reported previously;^29^ however, the increased number of domains (multilamellar vesicles) at high NaCl concentrations has not been evidenced, yet. This will be a future work by means of, e.g., scattering techniques. Figure 7 summarizes the two factors suggested here. The domain-to-domain interactions should be considered in a whole range of NaCl concentrations. However, the change in the amount of excess water made large impact on the viscosity change at low NaCl concentrations. We assumed, therefore, that the impact of the domain-to-domain interactions was limited at low NaCl concentrations.

## Conclusions

We studied the effects of inorganic salts on the rheological properties of α-gels (α-form hydrated crystals). The increased NaCl concentration resulted in a decreased viscosity in the [NaCl] range of 0–200 mmol dm^–3^, and afterward, the viscosity increased in the [NaCl] range of 200–1000 mmol dm^–3^. We suggest that the following two factors affected the viscosity. The first factor was the amount of excess water. The increased NaCl concentration resulted in a decreased *d*-spacing as a result of the screening of the electrostatic repulsion between the bilayers. This led to an increased amount of excess water, decreasing the viscosity up to 200 mmol dm^–3^. The second factor was the domain-to-domain interactions. A further increase in NaCl concentration resulted in the decreased electrostatic repulsion between the domains and/or the increased number of multilamellar vesicles. These effects contributed to the increased domain-to-domain interaction, increasing the viscosity in the [NaCl] range of 200–1000 mmol dm^–3^. The increased cohesion between the domains at high NaCl concentrations led to difficulty in recovering the original network structure once the network structure collapsed under the strain.

Cream products formulated by the α-gel generally include salts as viscosity modifiers, and their rheological properties should be controlled with good texture. Furthermore, the presence of electrolytes on the skin and the hardness of water affects the formulation and texture of such cream products. The findings of this study are expected to be useful for such applications.

## Supporting Information Available

(Figure S1) Optical and polarized microscope images obtained for the α-gel samples prepared at different NaCl concentrations and (Figure S2) static viscosities measured at [NaCl] = 100 and 800 mmol dm^–3^ (PDF)
