# Supplementary material for: Rheology of α-Gel Formed by Amino Acid-Based Surfactant with Long-Chain Alcohol: Effects of Inorganic Salt Concentration
Source: Langmuir. 2021 Jun 3;37(23):7032–8. doi: 10.1021/acs.langmuir.1c00626 (PMC8280742; doi:10.1021/acs.langmuir.1c00626)
Supplement: Supplementary file 1 — la1c00626_si_001.pdf [file la1c00626_si_001.pdf]

Supporting Information:

"Rheology of α-Gel Formed by Amino Acid-Based Surfactant with Long-Chain Alcohol: Effects of Inorganic Salt Concentration"

Kumika Ichihara,[1] Tadashi Sugahara,[1] Masaaki Akamatsu,[1] Kenichi Sakai,[1,2*] and Hideki Sakai[1,2]

Corresponding author e-mail: k-sakai@rs.tus.ac.jp

1. Department of Pure and Applied Chemistry, Faculty of Science and Technology, Tokyo University of Science
2. Research Institute for Science and Technology, Tokyo University of Science

Number of pages: 2
Number of figures: 2
Number of schemes: 0
Number of tables: 0

Table of Contents in Supporting Information

1. Methods
2. Results

1. Methods

Optical and polarized microscopy was performed using an Olympus IX73 microscope.

2. Results

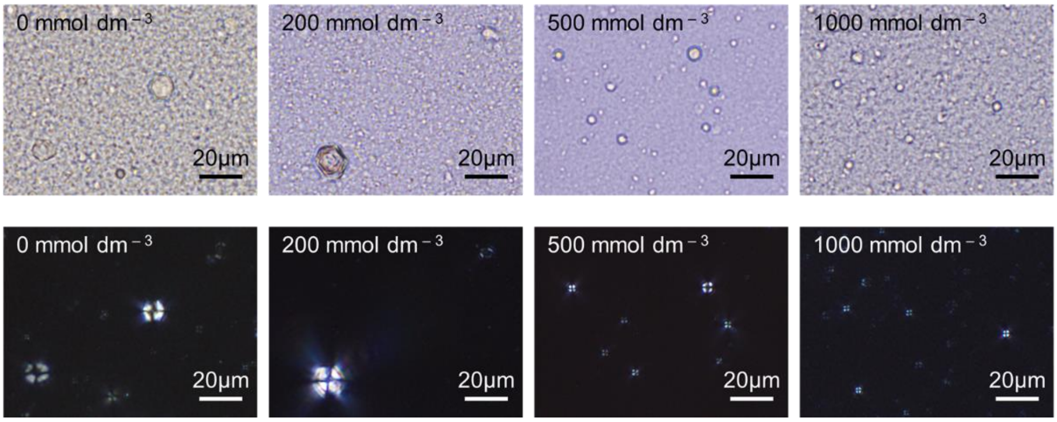

Figure S1. Optical and polarized microscope images obtained for the α-gel samples prepared at different NaCl concentrations. Vesicular or onion-like structures were observed in these images. The size of these domains did not change significantly within the resolution. The scale bars show 20 μm in length.

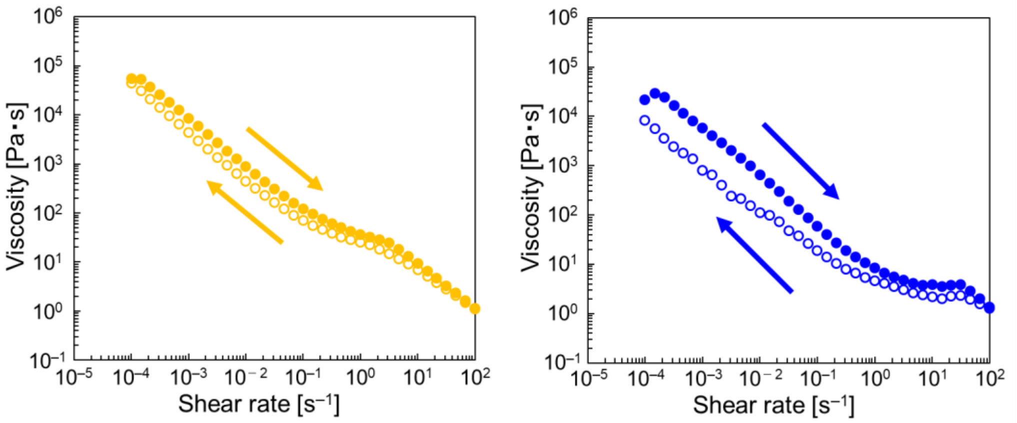

Figure S2. Static viscosity as a function of shear rate. The NaCl concentrations were varied at (left) 100, and (right) 800 mmol dm^{-3}. The filled circles correspond to the first shear rate sweep at increasing shear rates, while the empty circles correspond to the second shear rate sweep at decreasing shear rates.
